# Complete genome sequence of *Nitrosomonas europaea* W4 encoding Eut BMC for ethanolamine metabolism

**DOI:** 10.1128/mra.00172-25

**Published:** 2025-07-31

**Authors:** Hongfei Wang, Kai Liu, Jingxuan Deng, Jian Gao, Mingjun Liao

**Affiliations:** 1 Hubei Key Laboratory of Environmental Geotechnology and Ecological Remediation for Lake & River, Hubei University of Technology 47896, Wuhan, China; 2 Hengzhen Wuxi Biotechnology Co, Ltd, Wuxi, China; Indiana University, Bloomington, Bloomington, Indiana, USA

**Keywords:** *Nitrosomonas europaea*, Eut BMC, ethanolamine

## Abstract

The strain *Nitrosomonas europaea* W4 was isolated from a river water sample, and its complete genome sequence was determined. The genome encodes a functional Eut bacterial microcompartment for ethanolamine metabolism. Deciphering this genomic information is essential for optimizing high-biomass fermentation and advancing its application in wastewater treatment.

## ANNOUNCEMENT

*Nitrosomonas europaea* W4 isolated from the Shiqi River in Zhongshan, China. *N. europaea* exhibits strong ammonia-oxidizing capability and wide environmental distribution, but the strain is difficult to isolate. Currently, only *N. europaea* ATCC 19718 has a complete genome in NCBI (1). This new Asian isolate may reveal genetic differences from the reference strain. The isolation of *N. europaea* W4 was performed using a 96-well plate method with inorganic liquid medium (2), cultured at 30°C and 130 rpm in the dark. Bacterial biomass was harvested by centrifuging at 10,000 rpm for 5 min. Genomic DNA was extracted using the Mag-MK Bacterial Genomic DNA Extraction Kit. DNA quality was verified via Qubit 4.0 (Thermo Fisher Scientific) for concentration measurement and pulsed-field gel electrophoresis (CHEF Mapper XA, Bio-Rad) to confirm fragment integrity (>50 kb) (3) High-quality DNA was used for library preparation and sequencing.

Whole-genome sequencing was performed using PacBio Sequel IIe sequencing and Illumina NovaSeq6000 (Illumina Inc., San Diego, CA, USA). For Illumina sequencing, 1 µg of genomic DNA samples was fragmented into 400–500 bp segments using a Covaris M220 Focused Acoustic Shearer. Illumina sequencing libraries were prepared from the sheared fragments using the NEXTflex Rapid DNA-Seq Kit (Bioo Scientific, USA). For PacBio library preparation, 15 µg DNA was spun in a Covaris g-TUBE (Covaris, MA) at 6,000 RPM for 60 s using an Eppendorf 5424 centrifuge. The sequencing library was purified three times with 0.45 × volumes of Agencourt AMPure XP beads. A ~10 kb insert library was prepared and sequenced on one SMRT cell using standard protocols. The quality control of Illumina data was performed using fastp (v.0.23.0) (4) with all software parameters set to their defaults. The PacBio Sequel IIe platform produced HiFi reads. The quality-controlled Illumina data and HiFi reads were assembled using Unicycler (v.0.4.8) (5). The actual annotation and visualization procedures are as follows: Predicted CDS were annotated against NR, Swiss-Prot, GO, COG, and KEGG databases using BLAST+ (v.2.3.0), Diamond (v.0.8.35). Annotation results were visualized with Artemis (v18.2.0) (6). PacBio sequencing yielded 41,266 long reads (total 339,733,834 bp), with an average read length of 8.2 kb, a maximum read length of 22.6 kb, an N50 of 8,670 bp, and ~120× coverage. The Illumina data had a read length of 7,922,322 (total 1,196,270,622 bp). The sequencing depth was 151-fold and Q20/Q30 values of 98.70%/96.32%, achieving ~390× coverage. After completing the assembly of the bacterial genome, the NextDenovo (v2.5.2) tool was used to automatically circularize the genome. Comparative genomic analysis was conducted using the *N. europaea* ATCC 19718 reference genome (NCBI). The whole-genome average nucleotide identity, calculated via BLASTN, was 97.7% (7). The genome of *N. europaea* W4 comprises a chromosome (2,892,991 bp) and a plasmid (55,583 bp) (Table 1).

**TABLE 1:** Genomic properties of N. europaea W4

Type	Name	GC%	Size (bp)	Average length (bp)	rRNAs	tRNAs	Other RNA	CDSs
Chromosome	-	51.26	2,892,991	952	3	41	60	2,716
Plasmid	-	52.84	55,583	828	-	1	-	55

Functional annotation using the eggNOG-mapper v2.1.1.2 database identified genes encoding the Eut bacterial microcompartment (8), highlighting the strain’s potential for ethanolamine metabolism (9). The in-depth study of the genomic information of *N. europaea* W4 is important for achieving high-biomass fermentation of this isolate and its application in wastewater treatment.

## Data Availability

The raw sequencing data and the assembled genome have been submitted to the NCBI GenBank database. The genomic sequence is publicly available. The BioSample number is SAM47145788, the BioProject number is PRJNA1229865, and The Illumina raw data accession number is SRX27835090, and the PacBio raw data accession number is SRX29463302. The accession number for the complete assembled genome is GCA_039905905.1.The accession number of the chromosome sequence is CP157069.1, and that of the plasmid sequence is CP157070.1.
